# Lupus Nephritis, Autoantibody Production and Kidney Outcomes in Males with Childhood-Onset Systemic Lupus Erythematosus

**DOI:** 10.3390/pediatric14020030

**Published:** 2022-05-06

**Authors:** Scott E. Wenderfer, Alvaro Orjuela, Mir Reza Bekheirnia, Maria Pereira, Eyal Muscal, Michael C. Braun, Marietta De Guzman

**Affiliations:** 1 Division of Nephrology, Department of Pediatrics, Baylor College of Medicine, Houston, TX 77030, USA; ahorjuel@texaschildrens.org (A.O.); bekheirn@bcm.edu (M.R.B.); mcbraun@texaschildrens.org (M.C.B.); 2 Texas Children’s Hospital, Houston, TX 77030, USA; mxpereir@texaschildrens.org (M.P.); emuscal@bcm.edu (E.M.); mmdeguzm@texaschildrens.org (M.D.G.); 3 British Columbia Children’s Hospital, Vancouver, BC V6H 3V4, Canada; 4 Division of Rheumatology, Department of Pediatrics, Baylor College of Medicine, Houston, TX 77030, USA

**Keywords:** child, outcomes, lupus erythematosus, male, nephritis

## Abstract

Childhood-onset systemic lupus erythematosus (cSLE) only represents 20% of all SLE patients, and males with SLE only represent 10%. To study this rare SLE subset, males diagnosed with cSLE over a 30-year period were identified. Organ involvement, autoantibody production, hypocomplementemia, and kidney biopsy findings were compared to cSLE females. Outcomes were assessed using SLE Disease Activity Index scores, Systemic Lupus International Collaborating Clinics/American College of Rheumatology Damage Index, and Childhood Arthritis and Rheumatology Research Alliance definitions for nephritis responsiveness. Of 95 males and 545 females with cSLE, 62% and 57% developed nephritis, respectively. Median age of cSLE onset was 14 years in both genders. Among males, 80% of non-Hispanic whites, 64% of blacks, 59% of Hispanics, and 50% of Asians developed nephritis. The prevalence of pure and mixed class V membranous nephritis was 33%. Median follow-up was 3.2 years (range 0.1–18). Complete kidney responses were seen in 70% after a median 24 months; however, relapse rates were 46%. Kidney disease flares were 56% nephritic and 44% proteinuric. Males and females with cSLE present with comparable rates and nephritis class. While overall and kidney response rates are favorable, kidney disease relapses are common among males.

## 1. Introduction

As is seen in a number of autoimmune disorders, systemic lupus erythematosus (SLE) occurs more commonly in females than males [1,2]. In general, the female to male ratio ranges from 8–15:1, but in pre-pubertal cohorts the ratios are lower (2–6:1) [2,3,4,5,6,7]. The genetic, environmental, and hormonal differences between male and female SLE patients may play differing roles dependent on the age of disease onset. Since most clinical studies of adult- and childhood-onset lupus do not analyze outcomes stratified by gender, most study results are likely biased towards findings that predominate in females.

Male-specific data on childhood-onset SLE (cSLE) is sparse and, as with many large adult SLE studies, is embedded within much larger gender-inclusive cohorts. Most pediatric gender-inclusive studies contain few male subjects, with large pediatric cohorts’ typically reporting data on fewer than two dozen male patients. Data on males with cSLE including clinical manifestations, disease severity scores, kidney disease rates, and outcomes are intriguing, despite the limited numbers of subjects.

The Philadelphia group noted a very high frequency of familial autoimmunity (73%) in their published cohort of 11 males with cSLE [8]; interestingly, this finding has not been addressed in other cSLE case series. Data on SLICC/ACR Damage Index scores in male cSLE is conflicting, as 17 males from Saudi Arabia were reported to have high index scores on presentation [9], while a similar study of 13 males with cSLE from Vancouver failed to support this finding [10]. Data from Tennessee (*n* = 5) [3], India (*n* = 14) [11], Toronto (*n* = 15 males) [12], and South Korea (*n* = 22) [13] all indicate that males have a very high frequency of diffuse proliferative glomerulonephritis (DPGN) (38–100%, relative risk 2.9–3.9 compared to females with cSLE) and poor overall kidney outcomes. The problematic nature of kidney disease in males with cSLE is further supported by data from the Italian Collaborative Study (*n* = 35 males) where male gender associated with a 58% higher risk of lupus nephritis (LN) flare by multivariate analysis [14]. Despite these reports, the relative risk for progression to end-stage kidney disease (ESKD) in males with cSLE remains controversial; a Saudi group reported a high risk of progression in their cohort [9], while the Vancouver group did not [10]; none of the 13 males progressed to ESKD versus 8% of females at 7 years median follow-up. Not only is our understanding of male cSLE limited by a paucity of numbers, but significant confounders (ethnic/racial/ancestral differences, center-specific treatment variation, and a lack of uniform clinical reporting of disease scoring) make inter-center data comparisons problematic. While it may be argued that male cSLE is not distinct from female cSLE, data from the US Centers for Medicare and Medicaid Services (CMS) and the Renal Data System (USRDS) would suggest that this hypothesis is not correct, as the ratio of females-to-males shifts from 1:5 in those with all forms of lupus nephritis [7] to 1:4 in those who progress to ESKD [15]. The mechanistic basis for this apparent accelerated risk of ESKD in males with cSLE remains to be defined.

This study seeks to characterize the clinical features, natural history, and outcomes of male children diagnosed with SLE in the largest ever reported single center cohort using broadly based and accepted clinical criteria and disease activity indices in an ethnically diverse US patient population.

## 2. Materials and Methods

Study participants were identified from a cohort of children who were diagnosed with SLE before the age of 18 years of age at Texas Children’s Hospital between January 1986 and December 2019 (33 years). This study was approved by the Baylor College of Medicine Institutional Review Board and procedures followed were in accordance with the standards of the Helsinki Declaration of 1975/83. Patients were included if they fulfilled at least 4 of the ACR classification criteria for SLE [16]. LN was classified after kidney biopsy based on the 1995 modified WHO system [17]. Assigned gender was based on self-report at time of data abstraction. The clinical presentations and therapeutic responses of 7 male patients with proliferative LN [18] and 8 with pure membranous LN [19] have been published previously.

Antinuclear antibodies (ANA) and anti–double stranded DNA Abs (anti-dsDNA) were measured by indirect immunofluorescence using Hep-2 cells and *Crithidia luciliae* assay, respectively; ANA titer of more than 1:40 and an anti-dsDNA Ab titer of more than 1:10 or >50 IU/mL was considered positive. Use of different laboratories over the years with non-standardized detection methods dsDNA reactivity precluded our ability to study dsDNA titer as a quantitative characteristic. ANA profiles were routinely performed by Missouri labs and included anti-Ro/SSA, anti-La/SSB, anti-Sm, anti-RNP, anti-Scl70, and Jo-1 specificities [20]. Anti-phospholipid (aPL) Abs documented included IgG and IgM isotypes of anti-cardiolipin (aCL) and lupus anticoagulant (LAC). Positivity for LAC was assessed via a combination of methods including, a PTT-LA dilute Russell viper venom times (dRVVT) and Staclot assays [21]. SLE disease activity was calculated using the SELENA Systemic Lupus Erythematosus Disease Activity Index (SLEDAI) [22]. Organ damage was recorded using the SLICC/ACR (Systemic Lupus International Collaborating Clinics/American College of Rheumatology Atlanta, GA, USA) Damage Index [23].

Patients were treated at the discretion of their primary pediatric rheumatologist and nephrologist without the utilization of a specific protocol. Patients with proliferative LN (with or without mixed membranous lesions) tended to receive intravenous cyclophosphamide for 6–9 months, along with oral corticosteroids (±intravenous steroids), and hydroxychloroquine. Patients with pure membranous lesions tended to receive oral steroids and hydroxychloroquine, ±intravenous steroids, azathioprine, or mycophenolate mofetil. All patients received some form of maintenance immunosuppression after completion of induction therapy. Additional agents were added for refractory disease. Due to the heterogeneity of the therapies provided, data analysis was limited to the numbers of immunomodulatory drugs prescribed at each time point.

Kidney disease outcomes were assessed using the definitions published by CARRA in the consensus treatment plans for proliferative LN in cSLE [24]. A substantial response/complete remission was defined by (1) inactive urine sediment with <5 WBC/high powered field (hpf), <5 RBCs/hpf, and no casts; (2) urine protein to creatinine (UPC) ratio < 0.2; and (3) normal kidney function (Schwartz glomerular filtration rate (GFR) > 90 mL/min/1.73 m^2^). A moderate response was defined as at least 50% improvement in 2 of 3 core kidney disease parameters (kidney function, urine sediment, and proteinuria, max UPC ≤ 1.0) without clinically relevant worsening of the remaining core parameter. A mild response was defined as a 30–50% improvement in 2 of 3 core kidney parameters without clinically relevant worsening of the remaining core parameter. As a second method for assessing kidney outcomes, the Renal-SLEDAI was calculated as the total of all kidney components used to calculate the SLEDAI (hematuria, pyuria, proteinuria, and urinary casts) [25].

Kidney disease relapses or flares were classified as either proteinuric or nephritic, based on CARRA consensus definitions [24]. A proteinuric flare was defined as a persistent increase in proteinuria by UPC to values >0.5 after achieving a complete LN remission, or a doubling of proteinuria with UPC > 1 after achieving a partial response. A nephritic flare was defined as an increase in hematuria or recurrence of active urinary sediment (based on analysis of spun urine by a pediatric nephrologist), with or without a concomitant increase in proteinuria. The occurrence of a kidney disease flare did not require an elevation in serum creatinine. Hypertension was defined as elevation of blood pressure above 95th percentile for age, gender and height on three separate occasions.

Data were analyzed with SigmaPlot software (version 11.0, Inpixion, Palo Alto, CA). Frequencies and percentages were calculated for categorical variables; medians and interquartile ranges (IQR), for continuous variables. Fisher exact analysis was performed for non-parametric categorical variables. The Mann-Whitney U test was utilized for all analyses of continuous non-parametric variables. Kidney disease outcomes for males with cSLE were assessed by Kaplan–Meier analysis (time to response and/or flare). A *p*-value < 0.05 was considered statistically significant.

## 3. Results

Ninety-five (95) male cSLE patients were identified in the TCH cSLE cohort (Table 1). This excludes 3 patients (2 Hispanic, and 1 Caucasian) with isolated cutaneous lupus, one (Hispanic) with isolated LAC hypoprothrombinemia syndrome, and one (Hispanic) with minocycline-induced lupus. The median age of onset (defined as the time when the patient first met at least 4 ACR classification criteria for SLE) was 14 years of age (range 2–17). Of the 95 patients, most were Hispanic, but race/ethnicity varied widely as also seen in female cSLE patients.

**Figure 1:**
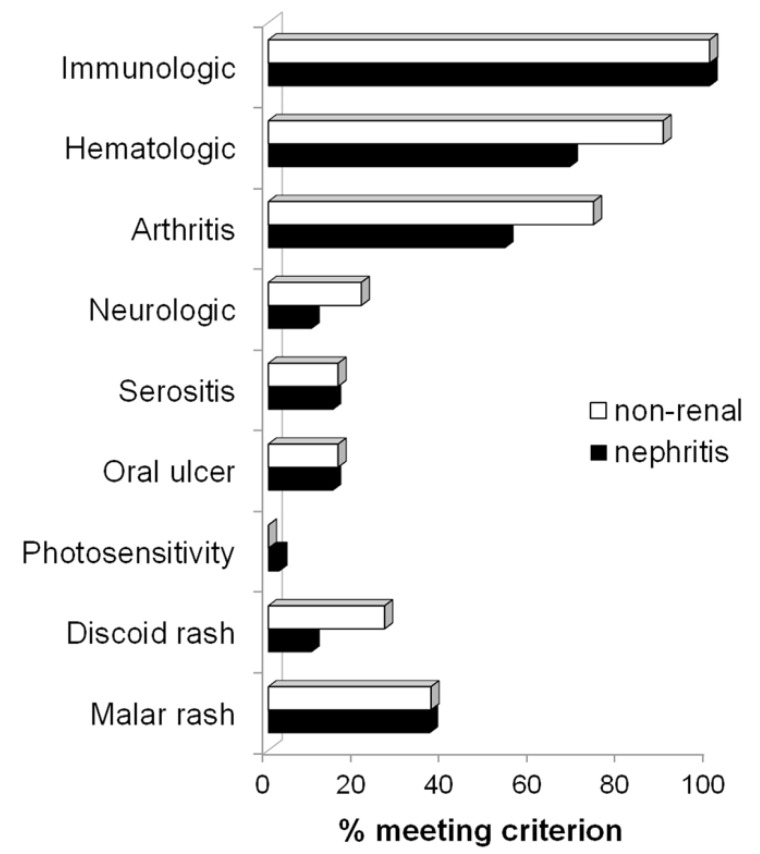
**Proportion of ACR classification criteria met by males with childhood-onset SLE with and without nephritis.** Patients who developed kidney involvement at any time during follow-up (biopsy not required, *n* = 59, black) were compared with those who never met criteria for kidney disease (based on ACR classification, *n* = 36, white bars). All patients tested positive for antinuclear antibodies. ACR = American College of Rheumatology. *p*-values < 0.05 for each comparison between lupus nephritis and non-renal subsets.

ACR Classification Criteria met for subjects in the cohort are listed in Figure 1. There were no significant differences in rates of extra-renal manifestations in boys with or without kidney involvement. Males with cSLE and LN had slightly higher rates of immunologic disorder (95% vs. 89%) and discoid rash (11% vs. 8%), and slightly lower rates or arthritis (51% vs. 64%), serositis (15% vs. 20%), and oral ulcers (15% vs. 20%), but none of these differences were statistically significant.

Additional manifestations of SLE in our male cohort include macrophage activation syndrome/secondary hemophagocytic lymphohistiocytosis (*n* = 3), autoimmune hepatitis (*n* = 2), aPL syndrome (*n* = 1), LAC hypoprothrombinemia syndrome (*n* = 1), and thrombotic microangiopathy (*n* = 1). The presumptive initial diagnosis in 14 boys who later met criteria cSLE was idiopathic thrombocytopenic purpura (*n* = 8), mixed connective tissue disease (*n* = 5), and Langerhans cell histiocytosis (*n* = 1). Additional autoimmune features were described in 17 patients and included: Sjogren’s syndrome (*n* = 8), non-specific colitis (*n* = 3), inflammatory bowel disease (*n* = 1), psoriasis (*n* = 1), thyroiditis (*n* = 1), reactive airway disease (*n* = 1), leukocytoclastic vasculitis (*n* = 1), erythema nodosum (*n* = 1), and Tourette’s (*n* = 1).

**Figure 2:**
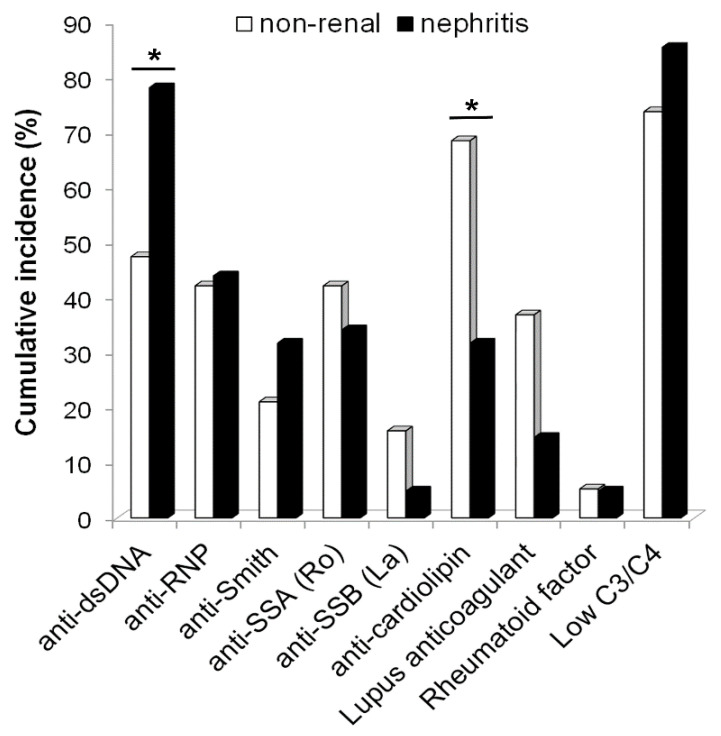
**Proportion of males with childhood-onset SLE with and without nephritis who tested positive for specific autoantibodies or hypocomplementemia.** Patients who developed kidney involvement at any time during follow-up (black, *n* = 59) were compared with those who never met criteria for kidney disease (white bars, *n* = 36). Cumulative incidence is shown, which includes testing positive at baseline or anytime during follow-up. * *p*-values < 0.05 for comparison between lupus nephritis and non-renal subsets.

The cumulative incidence rates of auto-Ab reactivities in male cSLE include 68% for anti-dsDNA, 43% for anti-RNP, 28% for anti-Smith, 37% for anti-Ro, 8% for anti-SS-B (La), and 5% for rheumatoid factor (Figure 2). One patient had anti-platelet, 4 had anti-thyroglobulin, 2 had anti-thyroid peroxidase, and 1 had anti-Scl70 Abs, but these were not routinely tested in all patients. The cumulative incidence of developing aPL Abs was 60% in our male cSLE cohort. Of those 36 patients, the proportions testing positive for LAC, aCL Abs or both were 36%, 69%, and 10%. The cumulative incidence of hypocomplementemia was 82%.

Cumulative incidence of kidney disease was 62% in males and 58% in females with cSLE (Table 2). A total of 90% of both males and females with cSLE presented with LN, and the remainder developed LN within 2 years of diagnosis. The median age of onset of LN in males with cSLE was 13 years of age (Table 1). Kidney disease was diagnosed in 80% of white, 64% of black, 59% of Hispanic, and 50% of Asian males. Comparatively, 56% of white, 60% of black, 57% of Hispanic, and 50% of Asian females with cSLE developed kidney disease. The rates of LN class II and III in males were higher and the rates of LN class IV were lower than in females with cSLE (*p*-value = 0.001). Of the 59 male cSLE patients with kidney disease, 78% tested positively for anti-dsDNA, 44% anti-RNP, 33% anti-Sm, 34% anti-Ro, 5% anti-La, 32% aCL Abs, and 15% tested positively for LAC (Figure 2).

On initial kidney biopsy, 21% of males and 13% of females had WHO class II lesions only (Table 2). Class III or IV lesions were seen in 41% of males and 43% of females; 20% and 15%, respectively, had mixed class III + V or VI + V lesions. Pure class V lesions were seen in comparable numbers of males and females with cSLE as well. Of males with LN, 14% had crescentic GN and 24% developed an elevated serum creatinine at time of biopsy (Table 3). Urinary abnormalities (proteinuria, proliferative urine sediment) were present at time of SLE diagnosis in 70%, and in all remaining patients with LN after a median 2 months (range 1–6 months) after cSLE diagnosis. Two patients presented with isolated lupus-like nephritis and only later met criteria for a diagnosis of SLE (1 month and 10 years after presentation, respectively).

Of the male cSLE patients with non-renal disease (*n* = 36), 50% were Hispanic, 22% black, 14% Asian, and 8% non-Hispanic white (Table 1, similar to distributions seen in female cSLE). Of non-renal cSLE males, 47% tested positively for anti-dsDNA, 42% anti-RNP, 20% anti-Sm, 42% anti-Ro, 16% anti-La and 68% aCL Abs, and 37% tested positively for LAC (Figure 2). The presence of anti-dsDNA Abs was significantly greater in LN compared to non-renal SLE (*p*-value = 0.035). Conversely, the presence of aCL Abs was significantly less common in those with kidney disease (*p*-value = 0.012).

There was a high degree of familial disease in our cohort (Table 1). Positive family histories of autoimmune disease were seen in 46% of males and 39% of females in our cohort. Seven percent of males with cSLE had a mother (*n* = 6) and or a father (*n* = 1) with SLE, compared to only 1.3% of females (all mothers, *p*-value < 0.0002). Two Asian boys and three girls (2 black, 1 Hispanic) had sisters with SLE. Five additional males and 18 females had one or more cousins with SLE (gender of cousin unavailable). In total, 15% of male and 5% of female cSLE patients had a close family member with SLE (*p*-value < 0.001). A range of autoimmune processes were seen in the other 31% of male cSLE patients with positive family histories, including (in order or prevalence) thyroid disease, rheumatoid arthritis, inflammatory bowel disease, multiple sclerosis, localized scleroderma, systemic sclerosis, and pemphigoid.

**Figure 3:**
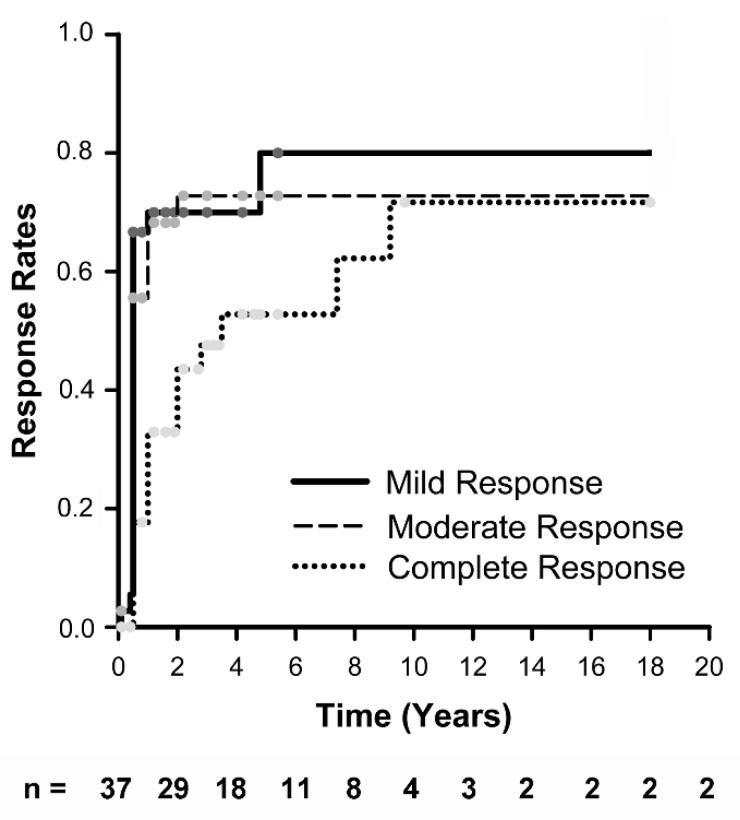
**Kaplan–Meier analysis of kidney responses.** Proportions of male patients with cSLE and nephritis having achieved at least mild (solid), moderate (dashed), and complete responses (dotted line) were plotted over time in years. The number of patients followed at each time point is listed beneath the plot. Response definitions are taken from the CARRA consensus treatment plans for childhood-onset lupus nephritis (23).

**Figure 4:**
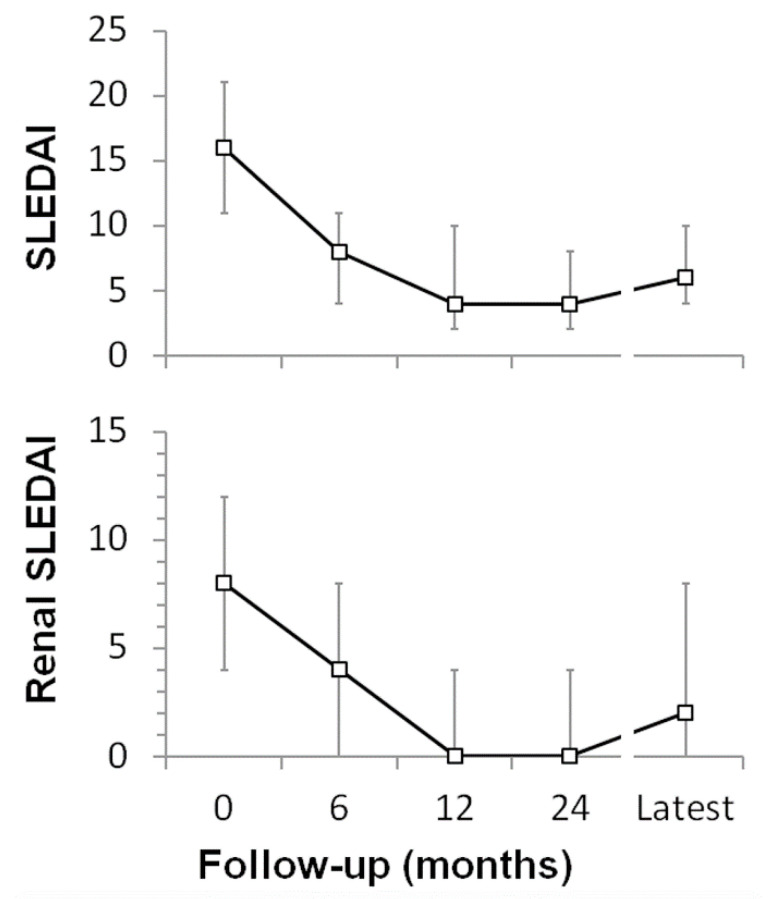
**Disease activity scores over time in males with childhood-onset SLE on therapy.** The Systemic Lupus Erythematosus Disease Activity Index (SLEDAI) score and Renal-SLEDAI score both were calculated for all patients (*n* = 41 at disease onset, *n* = 34 at 6 m, *n* = 29 at 12 m, *n* = 29 at 24 m, and 37 at latest follow-up). Data is shown as median score and error bars represent the intra-quartile ranges (IQR). Latest follow-up was a median of 3.4 years (IQR 1.6–6).

Kidney disease outcomes were available for 37 of the 59 males with cSLE and LN (Figure 3). Median time to mild or moderate response was 6 months, and median time to complete response was 2 years. The overall response rate to therapy was 71% at 12 months, which included 33% with complete and 27% with moderate response (Table 4). At a median follow-up of 3.5 years (IQR 1.6–6.2 years), 70% of males with cSLE and LN achieved complete kidney response. However, 9% of patients developed a kidney flare by 12 months, and 18 kidney flares were diagnosed in 22% of male cSLE patients at latest follow-up. Four males developed 2 kidney flares and three more developed 3 flares, for an annualized flare rate of 0.14 flares/person-year in males with cSLE and LN. Therefore, 62% were not in remission at latest follow-up, despite taking a median of 4 immunomodulatory drugs. There were equal numbers of proteinuric and nephritic flares. However, most lupus flares were mild. Overall, there was a decrease in median SLEDAI score and median Renal-SLEDAI score over time (Figure 4).

At latest follow-up (median 3.2 years, IQR 0.8–6 years), 1 male (secondary to infection) and 6 females with cSLE died (infection, pulmonary hemorrhage, and heart failure). Among survivors, the median SLICC/ACR Damage Index score (SDI) was zero (IQR 0–2, max 5, similar males versus females). Hypertension was still present in 62% of patients. As measured by glomerular filtration rate, there was no decline in kidney function in either gender over the duration of follow-up (Table 4). ESKD-free survival was 99% in males and 96% in females, and kidney function was preserved in over 75% of each.

## 4. Discussion

We report data for one of the largest single center, all male cohorts of childhood-onset SLE in the United States. The findings support those from smaller gender-specific cSLE case series: in males with cSLE, kidney disease presents early in the disease course and is often severe. The nephritis in these patients is responsive to therapy, but often takes 2–4 years and multiple medications to achieve remission. While progression to ESKD was the exception, our long-term follow-up data suggest that male cSLE patients suffer a high rate of kidney disease relapse. These results have important implications for the management of boys with cSLE.

In our cohort, the median age of onset was 14 years (range 2–17). While our ethnic/racial distribution (46% Hispanic, 23% black, 16% white, and 11% Asian) differs significantly from three other cohort studies on effects of gender (Toronto [12], Vancouver [10], and Saudi Arabia [9]), it is most reflective of the current ethnic/racial distribution of cSLE in the United States [6,7]. The degree of familial disease in our cohort is comparable to the frequency of familial autoimmunity reported in other US based cSLE studies [8,26]. However, the rates in males with cSLE (46% with autoimmunity and 15% with SLE) were significantly higher.

Our study also extends previously reported adult data on the relationship between gender and auto-Ab production. In our cohort, the high cumulative incidence of anti-cardiolipin Abs and the low frequency of developing anti-Ro Abs resembled rates reported in adult males [27,28,29]. The cumulative incidence of anti-dsDNA and anti-aPL Abs is similar to the rates reported in most gender-inclusive cSLE cohorts [11]. The findings that the rates of aCL and lupus anticoagulant were higher in males with cSLE without LN were surprising. No difference has been reported in aSLE populations with and without LN [30,31]. Unfortunately, we did not have available data on other aPL Abs such as anti-beta2 glycoprotein I IgG or IgM. The percentage of our patients presenting with hypocomplementemia (86%) does not, however, mirror rates reported in adult male cohorts (47 to 61%) [28,29,32]. However, the rates of discoid lesions, serositis, or thrombosis in our cohort were significantly lower than those reported in males with adult-onset SLE [1,2], and we failed to reproduce data in the literature suggesting significant differences in extra-renal lupus manifestations based on gender.

The incidence of kidney involvement at disease onset and the cumulative incidence of kidney disease in our male cSLE cohort is not significantly different from the rates reported in gender-inclusive cSLE cohorts [4,5,27,33]. Although not statistically significant, data trends suggest there are possible racial/ethnic differences in LN prevalence rates: 80% of white, 64% of black, 59% of Hispanic, and 50% of Asian males with cSLE developed LN. While the predilection for LN in Hispanic and black males with cSLE is consistent with previously published data, our data suggests, that rates of LN in white males may be higher and rates in Asian males may be lower than previously reported [7,27,34]. Compared to data from Toronto, Tennessee, and India there were fewer male cSLE patients in our cohort with class II or class IV lesions, and more with class III and class V [3,11,12]. Our data suggest that boys with cSLE do not have a preponderance of DPGN lesions. The large numbers of pure class V and mixed proliferative/membranous forms of LN seen in our boys with cSLE may reflect a geographic or ethnic phenomenon, as a similar distribution of LN class has been reported previously in gender-inclusive cohorts from the Southeastern U.S [19,35].

There was a decrease in overall extra-renal disease activity, as monitored by SLEDAI scores over the first 24 months, and a majority of our male cSLE patients had a final SLICC/ACR Damage Index score of zero. Damage scores were lower than those reported for adult male SLE cohorts [28] and the Vancouver cSLE cohort, where 54% of males had SDI ≥ 2 after 7 years follow-up [10]. This might be due to improvements in disease management over the past 2 decades. Despite the improvements in extra-renal disease, our data indicates that males in our cohort with cSLE have a prolonged LN course, which is consistent with male-specific data from adult SLE studies [27,36,37].

To assess LN responses, three methods were utilized. First, kidney disease responses and relapses were analyzed using CARRA response definitions [24]. Mild and moderate LN responses were achieved in 49% of patients by 6 months; however, the median time to complete response was 2 years. The overall rate of sustained response to therapy was only 60%, with only 38% of male patients sustaining a complete LN response. Nearly 10% of males developed an early LN flare (within 12 months of diagnosis) and overall, 22% flared in less than 4 years of follow-up. This tended to be after steroid withdrawal but prior to withdrawal of other immunosuppression (data not shown). There were roughly equal numbers of proteinuric and nephritic flares. Males with cSLE also had a high rate of LN flare in the Italian Collaborative Study [14]. In aggregate, 62% of the patients in our cohort had active kidney disease at last follow-up, despite receiving a median of 4 immunomodulatory drugs.

Second, the Renal-SLEDAI score was assessed. Median scores decreased 50% by 6 months and 100% by 12 months. Due to LN flares, the final median kidney-SLEDAI for the cohort was 2, which was 25% of the score at initial presentation. Renal-SLEDAI score is based on presence of hematuria, pyuria, proteinuria, and urinary casts, with each finding assigned 4 points for a maximum of 16 points. The CARRA response definitions include only proteinuria and urinary casts, but also includes kidney function which is not in the SLEDAI. In our male cSLE cohort, both methods for assessing outcome provide similar findings. However, median kidney-SLEDAI scores suggest that the persistent kidney disease activity in our male cSLE cohort at latest follow-up was fairly modest.

Third, kidney survival and estimated GFR at last follow-up was analyzed. ESKD-free survival was 99% at last follow-up and kidney function was preserved in over 75% of the patients. However, the low level of activity suggested by Renal-SLEDAI score and the low levels of damage suggested by the SLICC/ACR Damage Index did not capture the decreased kidney function seen in 25% of males with cSLE. As with any currently available outcome measure, it is not possible to distinguish between proteinuria from persistent disease activity and proteinuria from irreversible kidney scarring. Overall, our findings were more aligned with the favorable outcomes reported by the Vancouver and Italian groups [10,14] than the unfavorable outcomes reported by others [3,8,9,12,13].

Limitations of this study include its retrospective nature, changes in practice patterns and biopsy classification schemes over time, and the heterogeneity of SLE itself. Gender was self-reported at time of data abstraction: biologic sex was not available, nor was data available on rates of trans or non-conforming youth within the cohort: unfortunately, this data is not accurately captured in electronic health systems. It was not possible to factor in the effects of either the efficacy of specific immunomodulatory therapy or treatment non-adherence. Quantitative data on complement C3 and C4 levels and dsDNA titer from a central laboratory was not available for regression modeling. Kidney disease outcome data was only available for half of the male cSLE patients and not available for the females. But rates of patient and ESKD-free survival was available for females with cSLE, as were baseline demographic and clinical information as well as lupus nephritis classification data. Strengths include the large cohort size, utilization of consensus childhood-specific outcome definitions, and the uniquely diverse racial/ethnic population in the Texas gulf coast.

## 5. Conclusions

The findings of this cohort study support the hypothesis that, SLE in males under the age of 18 years manifest disease with early and severe kidney involvement, a high frequency of anti-dsDNA and anti-cardiolipin Abs, and high rates of kidney disease relapse. Despite this, at least prior to transitioning their medical care from pediatric to internal medicine hospitals/clinics, very few male patients with cSLE progressed to ESKD.

## Figures and Tables

**Table 1:** Demographics and clinical history of 95 males and 545 females with childhood-onset SLE, with and without kidney involvement.

ALL cSLE	Males (*n* = 95)		Females (*n* = 545)	
Age at SLE diagnosis, median, (IQR *)	14	(11–15)	14	(11–16)
Ethnicity, *n* (%)				
Hispanic	44	(46)	212	(39)
Non-Hispanic White	15	(16)	84	(15)
Asian	10	(11)	48	(9)
Black	22	(23)	179	(33)
Mixed	1	(1)	16	(3)
Unknown	3	(3)	6	(1)
Family history of autoimmune disease	44	(46)	215	(39)
Family history of SLE, *n* (%)	27	(28)	105	(19)
**cSLE with LN Ever**	**Males (*n* = 59)**		**Females (*n* = 314)**	
Age at SLE diagnosis, median, (IQR *)	13	(11–14)	14	(11–15)
Ethnicity, *n* (%)				
Hispanic	26	(44)	121	(39)
Non-Hispanic White	12	(20)	47	(15)
Asian	5	(8)	24	(8)
Black	14	(24)	107	(34)
Mixed	0	(0)	11	(4)
Unknown	2	(3)	4	(1)
Family history of autoimmune disease	27	(46)	108	(34)
Family history of SLE, *n* (%)	17	(29)	50	(16)
**Non-renal cSLE**	**Males (*n* = 36)**		**Females (*n* = 231)**	
Age at SLE diagnosis, median, (IQR *)	13	(11–16)	13	(10–14)
Ethnicity, *n* (%)				
Hispanic	18	(50)	91	(39)
Non-Hispanic White	3	(8)	37	(16)
Asian	5	(14)	24	(10)
Black	8	(22)	72	(31)
Mixed	1	(3)	5	(2)
Unknown	1	(3)	2	(1)
Family history of autoimmune disease	17	(47)	107	(46)
Family history of SLE, *n* (%)	10	(28)	55	(24)

* IQR = interquartile range. *p*-values > 0.05 for each comparison between LN (lupus nephritis) and non-renal subsets.

**Table 2:** WHO class in *n* = 59 males and *n* = 314 females with cSLE and lupus nephritis *.

Feature	Males, *n* (%)	Females, *n* (%)
WHO Class		
Class I	1 (2)	11 (4)
Class II	11 (19)	27 (9)
Class III	14 (24)	60 (19)
Class IV	10 (17)	75 (24)
Class V	9 (15)	54 (17)
Mixed class III + V	6 (10)	22 (7)
Mixed class IV + V	6 (10)	26 (8)
Class VI	0 (0)	1 (0.3)
Not biopsied/unknown	2 (3)	38 (12)

* *p*-Value > 0.05 for LN classification between male and female cSLE cohorts.

**Table 3:** Histopathology and clinical manifestations of kidney injury at time of diagnosis in *n* = 59 males with lupus nephritis.

Feature	Males, *n* (%)
Histopathology	
Mesangial hypercellularity	53 (90)
Cellular crescents	8 (14)
Endocapillary proliferation	12 (24)
Membranous lesions	28 (48)
Clinical manifestation *	
Elevated serum creatinine (*n* = 37)	9 (24)
Nephritis (*n* = 37)	30 (81)
Nephrotic syndrome (*n* = 35)	15 (35)
Hypertension (*n* = 36)	17 (47)

* Clinical manifestations only available for subset of male LN patients.

**Table 4:** Outcomes of males with childhood-onset lupus nephritis.

	Onset (*n* = 37)	6 mos (*n* = 36)	12 mos (*n* = 33)	24 mos (*n* = 34)	Latest F/U * (*n* = 37)
Responders (%) ^§^	0%	66%	63%	61%	49%
Complete response	0	17%	33%	35%	38%
Moderate response	0	35%	27%	18%	19%
Mild response	0	14%	3%	0%	3%
Renal Flares (%) ^§^	0%	0%	9%	21%	22%
Proteinuric (*n*)	0	0	2	3	3
Nephritic (*n*)	0	0	1	4	5
Median GFR (IQR) ^§§^	122 (103–135)	118 (102–139)	122 (106–147)	124 (94–135)	125 (90–140)
Immunomodulatory drugs ^¥^, median number (range)	1 (0–2)	3 (2–4)	4 (3–5)	4 (3–6)	4 (1–7)

* median follow-up was 3.4 years after onset of nephritis, intra-quartile range 1.6–6. Disease duration of SLE at onset of nephritis was a median of 0 years, range 0–6. Follow-up data not available for each participant at each time point. ^§^ Definitions taken from Consensus Treatment Plan for Proliferative Lupus Nephritis (Mina et al., 2012). ^§§^ IQR = intraquartile range. ^¥^ Drugs include azathioprine, corticosteroids, cyclophosphamide, cyclosporine, intravenous immunoglobulin, hydroxychloroquine, methotrexate, mycophenolate mofetil, and tacrolimus.

## Data Availability

Restrictions apply to the availability of these data. Data was obtained from patients at Texas Children’s Hospital and are available from the authors with the permission of Texas Children’s Hospital.

## References

[1] Lu L.J., Wallace D.J., Ishimori M.L., Scofield R.H., Weisman M.H. (2010). Review: Male systemic lupus erythematosus: A review of sex disparities in this disease. Lupus.

[2] Murphy G., Isenberg D. (2013). Effect of gender on clinical presentation in systemic lupus erythematosus. Rheumatology.

[3] Lau K.K., Jones D.P., Hastings M.C., Gaber L.W., Ault B.H. (2006). Short-term outcomes of severe lupus nephritis in a cohort of predominantly African-American children. Pediatr. Nephrol..

[4] Brunner H.I., Gladman D.D., Ibanez D., Urowitz M.D., Silverman E.D. (2008). Difference in disease features between childhood-onset and adult-onset systemic lupus erythematosus. Arthritis Rheum..

[5] Hersh A.O., von Scheven E., Yazdany J., Panopalis P., Trupin L., Julian L., Katz P., Criswell L.A., Yelin E. (2009). Differences in long-term disease activity and treatment of adult patients with childhood- and adult-onset systemic lupus erythematosus. Arthritis Rheum..

[6] Son M.B.F., Johnson V.M., Hersh A.O., Lo M.S., Costenbader K.H. (2014). Outcomes in Hospitalized Pediatric Patients With Systemic Lupus Erythematosus. Pediatrics.

[7] Hiraki L.T., Feldman C.H., Liu J., Alarcón G.S., Fischer M.A., Winkelmayer W.C., Costenbader K.H. (2012). Prevalence, incidence, and demographics of systemic lupus erythematosus and lupus nephritis from 2000 to 2004 among children in the US medicaid beneficiary population. Arthritis Rheum..

[8] Apenteng T., Kaplan B., Meyers K. (2006). Renal outcomes in children with lupus and a family history of autoimmune disease. Lupus.

[9] Al-Mayouf S.M., Al Sonbul A. (2008). Influence of gender and age of onset on the outcome in children with systemic lupus erythematosus. Clin. Rheumatol..

[10] Miettunen P.M., Ortiz-Alvarez O., Petty R.E., Cimaz R., Malleson P.N., Cabral D.A., Ensworth S., Tucker L.B. (2004). Gender and ethnic origin have no effect on longterm outcome of childhood-onset systemic lupus erythematosus. J. Rheumatol..

[11] Hari P., Bagga A., Mahajan P., Dinda A. (2009). Outcome of lupus nephritis in Indian children. Lupus.

[12] Celermajer D.S., Thorner P.S., Baumal R., Arbus G.S. (1984). Sex differences in childhood lupus nephritis. Am. J. Dis. Child..

[13] Lee B.S., Cho H.Y., Kim E.J., Kang H.G., Ha I.S., Cheong H.I., Kim J.G., Lee H.S., Choi Y. (2007). Clinical outcomes of childhood lupus nephritis: A single center′s experience. Pediatr. Nephrol..

[14] Ruggiero B., Vivarelli M., Gianviti A., Benetti E., Peruzzi L., Barbano G., Corona F., Ventura G., Pecoraro C., Murer L. (2013). Lupus nephritis in children and adolescents: Results of the Italian Collaborative Study. Nephrol. Dial. Transplant..

[15] Hiraki L.T., Lu B., Alexander S.R., Shaykevich T., Alarcon G.S., Solomon D.H., Winkelmayer W.C., Costenbader K.H. (2011). End-stage renal disease due to lupus nephritis among children in the US, 1995–2006. Arthritis Rheum..

[16] Hahn B.H., McMahon M.A., Wilkinson A., Wallace W.D., Daikh D.I., Fitzgerald J.D., Karpouzas G.A., Merrill J.T., Wallace D.J., Yazdany J. (2012). American college of rheumatology guidelines for screening, treatment, and management of lupus nephritis. Arthritis Care Res..

[17] Churg J., Bernstein J., Glassock R.J. (1995). Renal Disease: Classification and Atlas of Glomerular Diseases.

[18] Askenazi D., Myones B., Kamdar A., Warren R., Perez M., De Guzman M., Minta A., Hicks M.J., Kale A. (2007). Outcomes of children with proliferative lupus nephritis: The role of protocol renal biopsy. Pediatr. Nephrol..

[19] Sorof J.M., Perez M.D., Brewer E.D., Hawkins E.P., Warren R.W. (1998). Increasing incidence of childhood class V lupus nephritis. J. Rheumatol..

[20] ANA Complete Profile. https://muhealth.testcatalog.org/show/ANA-Comp).

[21] Pierangeli S.S., de Groot P.G., Dlott J., Favaloro E., Harris E.N., Lakos G., Ortel T., Meroni P.L., Otomo K., Pengo V. (2011). ‘Criteria’aPL tests: Report of a task force and preconference workshop at the 13th International Congress on Antiphospholipid Antibodies, Galveston, Texas, April 2010. Lupus.

[22] Petri M., Kim M.Y., Kalunian K.C., Grossman J., Hahn B.H., Sammaritano L.R., Lockshin M., Merrill J.T., Belmont H.M., Askanase A.D. (2005). Combined oral contraceptives in women with systemic lupus erythematosus. N. Engl. J. Med..

[23] Gladman D., Ginzler E., Goldsmith C., Fortin P., Liang M., Urowitz M., Bacon P., Bombardieri S., Hanly J., Hay E. (1992). Systemic lupus international collaborative clinics: Development of a damage index in systemic lupus erythematosus. J. Rheumatol..

[24] Mina R., von Scheven E., Ardoin S.P., Eberhard B.A., Punaro M., Ilowite N., Hsu J., Klein-Gitelman M., Moorthy L.N., Muscal E. (2012). Consensus treatment plans for induction therapy of newly diagnosed proliferative lupus nephritis in juvenile systemic lupus erythematosus. Arthritis Care Res..

[25] Singh S., Wu T., Xie C., Vanarsa K., Han J., Mahajan T., Oei H.B., Ahn C., Zhou X.J., Putterman C. (2012). Urine VCAM-1 as a marker of renal pathology activity index in lupus nephritis. Arthritis Res. Ther..

[26] Michel M., Johanet C., Meyer O., Frances C., Wittke F., Michel C., Arfi S., Tournier-Lasserve E., Piette J.C. (2001). Familial lupus erythematosus. Clinical and immunologic features of 125 multiplex families. Medicine.

[27] Mok C.C., Lau C.S., Chan T.M., Wong R.W. (1999). Clinical characteristics and outcome of southern Chinese males with systemic lupus erythematosus. Lupus.

[28] Tan T.C., Fang H., Magder L.S., Petri M.A. (2012). Differences between Male and Female Systemic Lupus Erythematosus in a Multiethnic Population. J. Rheumatol..

[29] Soto M.E., Vallejo M., Guillen F., Simon J.A., Arena E., Reyes P.A. (2004). Gender impact in systemic lupus erythematosus. Clin. Exp. Rheumatol..

[30] Parodis I., Arnaud L., Gerhardsson J., Zickert A., Sundelin B., Malmstrom V., Svenungsson E., Gunnarsson I. (2016). Antiphospholipid Antibodies in Lupus Nephritis. PLoS ONE.

[31] Loizou S., Samarkos M., Norsworthy P.J., Cazabon J.K., Walport M.J., Davies K.A. (2000). Significance of anticardiolipin and anti-beta(2)-glycoprotein I antibodies in lupus nephritis. Rheumatology.

[32] Garcia M.A., Marcos J.C., Marcos A.I., Pons-Estel B.A., Wojdyla D., Arturi A., Babini J.C., Catoggio L.J., Alarcon-Segovia D. (2005). Male systemic lupus erythematosus in a Latin-American inception cohort of 1214 patients. Lupus.

[33] Andrade R.M., Alarcon G.S., Fernandez M., Apte M., Vila L.M., Reveille J.D. (2007). Accelerated damage accrual among men with systemic lupus erythematosus: XLIV. Results from a multiethnic US cohort. Arthritis Rheum..

[34] Molina J.F., Drenkard C., Molina J., Cardiel M.H., Uribe O., Anaya J.M., Gomez L.J., Felipe O., Ramirez L.A., Alarcon-Segovia D. (1996). Systemic lupus erythematosus in males. A study of 107 Latin American patients. Medicine.

[35] Contreras G., Lenz O., Pardo V., Borja E., Cely C., Iqbal K., Nahar N., de La Cuesta C., Hurtado A., Fornoni A. (2006). Outcomes in African Americans and Hispanics with lupus nephritis. Kidney Int..

[36] Prete P.E., Majlessi A., Gilman S., Hamideh F. (2001). Systemic lupus erythematosus in men: A retrospective analysis in a Veterans Administration Healthcare System population. J. Clin. Rheumatol..

[37] Wang Y.F., Xu Y.X., Tan Y., Yu F., Zhao M.H. (2012). Clinicopathological characteristics and outcomes of male lupus nephritis in China. Lupus.

